# Neugeborenenscreening auf schwere kombinierte Immundefekte (SCID) in Deutschland

**DOI:** 10.1007/s00103-023-03773-6

**Published:** 2023-09-19

**Authors:** Sujal Ghosh, Michael H. Albert, Fabian Hauck, Manfred Hönig, Catharina Schütz, Ansgar Schulz, Carsten Speckmann

**Affiliations:** 1 Klinik für Kinder-Onkologie, -Hämatologie und Klinische Immunologie, Zentrum für Kinder- und Jugendmedizin, Universitätsklinikum Düsseldorf, Düsseldorf, Deutschland; 2 Kinderklinik und Kinderpoliklinik im Dr. von Haunerschen Kinderspital, Ludwig-Maximilians-Universität München, München, Deutschland; 3 Klinik für Kinder- und Jugendmedizin, Universitätsklinikum Ulm, Ulm, Deutschland; 4 Pädiatrische Immunologie, Klinik und Poliklinik für Kinder- und Jugendmedizin, Universitätsklinikum Carl Gustav Carus an der Technischen Universität Dresden, Dresden, Deutschland; 5 Pädiatrische Hämatologie und Onkologie, Zentrum für Kinder- und Jugendmedizin und Centrum für Chronische Immundefizienz, Institut für Immundefizienz, Medizinische Fakultät, Universitätsklinikum Freiburg, Freiburg, Deutschland; 6 Klinik für Kinder-Onkologie, -Hämatologie und Klinische Immunologie, Zentrum für Kinder- und Jugendmedizin, Universitätsklinikum Düsseldorf, Moorenstr. 5, 40225 Düsseldorf, Deutschland

**Keywords:** SCID, Angeborene Störungen der Immunität, Neugeborenenscreening, T‑Zellen, TREC, SCID, Inborn errors of immunity, Newborn screening, T cells, TREC

## Abstract

Patienten mit einem schweren kombinierten Immundefekt (SCID) erkranken aufgrund einer fehlenden bzw. gestörten T‑Zell-Immunität meist innerhalb der ersten Lebensmonate an schweren, oft letal verlaufenden Infektionen oder Zeichen der Immunfehlregulation. Nur durch die Korrektur des Immunsystems – in der Regel durch eine hämatopoetische Stammzelltransplantation (HSZT) – ist eine Heilung möglich. Pilotstudien und nationale Programme in den USA und Europa konnten zeigen, dass betroffene Kinder bereits im asymptomatischen Stadium durch das Neugeborenenscreening erkannt werden können. Dies ermöglicht es, Patienten mit SCID noch vor Auftreten schwerer Komplikationen zu behandeln, was den Erfolg der Therapiemaßnahmen wie HSZT erheblich verbessert.

Einem Bewertungsverfahren im Gemeinsamen Bundesausschuss (G-BA) folgend wurde 2019 auch in Deutschland ein Neugeborenenscreening auf SCID eingeführt. Die ersten Ergebnisse des Screenings (Trockenblutkarten von ca. 2 Mio. Neugeborenen im Zeitraum August 2019 bis Februar 2022) wurden vor Kurzem veröffentlicht. Neben klassischen SCID-Erkrankungen (Inzidenz 1:54.000) wurden, wie erwartet, auch Patienten mit einer syndromalen Grunderkrankung und T‑Zell-Lymphopenie identifiziert. Bei allen Patienten mit klassischem SCID wurde eine kurative Therapie geplant; 21 von 25 Patienten waren zum Zeitpunkt der Datenauswertung bereits transplantiert. Nur einer der 21 transplantierten Patienten verstarb an vorbestehenden Infektionen. Ein Vergleich des implementierten Screenings mit historischen Daten zeigt, dass das Screening in Deutschland erfolgreich umgesetzt wurde. Patienten mit SCID werden frühzeitig identifiziert und einer kurativen Therapie zugeführt.

## Abstract (en)

Patients with a severe combined immunodeficiency (SCID) harbor genetic mutations disrupting T cell immunity and hence suffer severe, life-threatening infections or manifestations of immune dysregulation within the first months of their life. The only cure is to correct their immune system, usually by means of hematopoietic stem cell transplantation (HSCT). Pilot studies and national programs in the United States and in European countries have shown that patients can be identified at an early asymptomatic stage through newborn screening. This allows treatment before the occurrence of severe complications, which improves the outcome of curative strategies like HSCT.

After assessment by the Federal Joint Committee (G-BA), the SCID screening was implemented into newborn screening in Germany in 2019. The first results of the screening (dry blood spot cards from around 2 million newborns between August 2019 and February 2022) were recently published. As expected, in addition to classic SCID diseases (incidence 1:54,000), infants with syndromic disorders and T cell lymphopenia were also identified. All patients with classic SCID were scheduled for curative treatment. Of the 25 patients with classic SCID, 21 were already transplanted at the time of data analysis. Only one of 21 transplanted patients died due to pre-existing infections. A comparison of the recent screening data with historical data suggests that SCID newborn screening has been successfully implemented in Germany. Patients with SCID are routinely identified very early and scheduled for curative therapy.

## Einleitung

Als schwerer kombinierter Immundefekt (*Severe Combined Immunodeficiency*, SCID) wird eine heterogene Gruppe angeborener Störungen der T‑Zell-Immunität bezeichnet. Ursächlich hierfür sind Mutationen in Genen, die essenziell für die Entwicklung bzw. Funktion von T‑Lymphozyten (nachfolgend „T-Zellen“) sind. Betroffene Kinder leiden an einem angeborenen schweren T‑Zell-Mangel und erkranken meist noch im Säuglingsalter an oftmals tödlich verlaufenden, opportunistischen Infektionen. Des Weiteren kann sich ein SCID auch durch Zeichen schwerer Immundysregulation, wie Entzündung der Haut oder des Darms, manifestieren [1, 2]. Eine kurative Therapie ist nur durch einen „Austausch des defekten Immunsystems“ mittels zelltherapeutischer Maßnahmen möglich. Das Routineverfahren hierzu ist die hämatopoetische Stammzelltransplantation (HSZT), die mittlerweile über ein halbes Jahrhundert bei SCID-Patienten mit Erfolg durchgeführt wird [3]. Zudem gibt es bei einzelnen SCID-Erkrankungen (z. B. bei Mangel an Adenosindesaminase (ADA) oder bei einem Defekt im Interleukin-2-Rezeptor-Gamma-Gen) auch die Möglichkeit einer Gentherapie. Hierbei werden Betroffenen zunächst Blutstammzellen entnommen. Diese werden im Labor *in vitro* durch gentechnologische Verfahren „repariert“, d. h., eine korrigierte Version des entsprechenden fehlenden bzw. fehlkodierten Gens wird eingebracht. Wie bei einer allogenen HSZT, bei der die Patienten Blutstammzellen gesunder Spender (Familien- oder Fremdspender) erhalten, erfolgt anschließend eine Infusion der „gentherapierten“ Patientenzellen. Im Regelfall erhalten die Patienten bei beiden Therapieverfahren vorab eine Chemotherapie, um das langfristige Anwachsen der Blutstammzellen im Knochenmark zu verbessern. Diese Gentherapie für SCID-Varianten ist aktuell in Deutschland nicht verfügbar [4, 5].

Neben einer „klassischen“ SCID-Erkrankung (primäre Entwicklungs- oder Funktionsstörung von T‑Zellen) können auch Thymusanlagedefekte mit einem schweren T‑Zell-Mangel und dem klinischen Bild eines SCID einhergehen. Diese treten oftmals im Rahmen von syndromalen Erkrankungen wie dem Mikrodeletionssyndrom 22q11.2 auf. Eine HSZT ist bei diesen Patienten nicht kurativ. Es besteht aber die Möglichkeit, diese Patienten mittels Transplantation von Thymusgewebe von gesunden Fremdspendern zu behandeln („allogene Thymustransplantation“). Diese hochspezialisierte Therapieform wird derzeit nur an 2 Standorten weltweit angeboten (Duke University in Durham, North Carolina, USA, und Great Ormond Street Hospital in London, Vereinigtes Königreich). In London steht die Therapie Patienten aus Deutschland aktuell auch noch nach dem Austritt des Vereinigten Königreichs aus der Europäischen Union (EU) zur Verfügung [6, 7].

Neben den vorgenannten Ursachen primärer T‑Zell-Mangelerkrankungen sind ferner auch sekundäre T‑Zell-Lymphopenien bei Neugeborenen differentialdiagnostisch zu berücksichtigen. Hiervon betroffen sind vor allem Säuglinge, die Lymphozyten aufgrund neonatologischer oder anatomischer Komplikationen wie Hydrops fetalis, Malformationen von Lymphgefäßen, Chylothorax, schweren Herzfehlern oder sonstigen Flüssigkeitsverlusten in den „dritten“ Raum verlieren und dadurch eine sekundäre Immundefizienz aufweisen. Ebenso kann die maternale Einnahme bestimmter Immunsuppressiva in der Schwangerschaft mit einer (im Regelfall vorübergehenden) erniedrigten T‑Zell-Zahl im Säuglingsalter vergesellschaftet sein [8, 9].

Der hohe klinische Stellenwert einer frühzeitigen Erkennung eines schweren angeborenen T‑Zell-Mangels ist in mehreren nachfolgend vorgestellten Studien gut belegt. Der Therapierfolg der oben genannten kurativen Ansätze korreliert stark mit der zum Zeitpunkt der Transplantation vorliegenden Krankheitslast. Bereits 2011 zeigte eine retrospektive Studie mit 108 an SCID erkrankten Säuglingen am Great Ormond Street Hospital in London einen Vorteil, diese Patienten früh zu identifizieren. Während das Gesamtüberleben der „Initialkohorte“ (d. h. Patienten, die im Rahmen einer klinischen Problematik diagnostiziert wurden, *n* = 48) nur 40 % betrug (Langzeitüberleben 19/48 Patienten), war der Verlauf bei den Geschwistern dieser Patienten, die ebenfalls eine SCID-Erkrankung hatten, deutlich besser. Da die genetische Prädisposition in der Familie schon bekannt war, konnte die Erkrankung direkt nach der Geburt diagnostiziert werden. Der Großteil der „Initialkohorte“ verstarb bereits vor der HSZT an Infektionen. In der Geschwisterkohorte zeigten sich nur wenige fatale Verläufe nach der HSZT; über 90 % (54/60 Patienten) dieser früh diagnostizierten Kinder überlebten [10].

Ebenfalls wurde durch eine nordamerikanische multizentrische retrospektive Studie aus den 2000er-Jahren der Einfluss von Alter und Infektionsstatus auf das Langzeitüberleben verdeutlicht. In der Datenerhebung, die insgesamt 240 Patienten umfasst, hatten Kinder, die im Alter von unter 3,5 Monaten ohne vorbestehende Infektion transplantiert wurden, ein Langzeitüberleben von über 90 %. Säuglinge mit einem Lebensalter von > 3,5 Monaten und bestehender Infektion zum Zeitpunkt der HSZT hatten ein Langzeitüberleben von nur noch etwa 50 % [11]. Auch in einer europäischen multizentrischen Studie gibt es ähnliche Ergebnisse. 338 Patienten mit SCID wurden hier zwischen den Jahren 2006 und 2014 untersucht und eine vorbestehende Infektion (bei 138 von 338 Patienten) war mit einem deutlich schlechteren Outcome assoziiert (2-Jahres-Überleben: 73 % vs. 87 % bei Patienten ohne Infektion; [3]).

Zusammenfassend kann unter anderem durch die oben genannten Studien gezeigt werden, dass bei Kindern, die früh und vor Eintreten von kritischen Organschäden diagnostiziert und mit prophylaktischen Maßnahmen stabilisiert wurden, ein signifikant besseres Behandlungsergebnis erreicht wird. Eine Früherkennung betroffener Kinder, idealerweise der gesamten Geburtskohorte, sollte daher angestrebt werden.

Seit 2008 wurden vor allem in den USA initial im Rahmen von regionalen Pilotstudien Neugeborene auf SCID gescreent. Erste Ergebnisse aus Kalifornien und Wisconsin zeigten bereits Mitte der 2010er-Jahre Erfolge: Patienten mit SCID konnten früh diagnostiziert und einer zügigen Behandlung (Infektionsprophylaxe, Zelltherapie) zugeführt werden [12–15].

In Deutschland wurde auf Initiative der Patientenorganisation Deutsche Selbsthilfe Angeborene Immundefekte (DSAI) und den medizinischen Fachgesellschaften Arbeitsgemeinschaft Pädiatrische Immunologie (API), Deutsche Gesellschaft für Kinder- und Jugendmedizin (DGKJ) und Deutsche Gesellschaft für Neugeborenenscreening (DGNS) schließlich 2014 der Antrag des Spitzenverbandes der Gesetzlichen Krankenversicherung (GKV) auf Bewertung eines SCID-Screenings im Rahmen des erweiterten Neugeborenenscreenings beim Gemeinsamen Bundesausschuss (G-BA) eingereicht. Im Rahmen des mehrjährigen Verfahrens erfolgte zunächst eine positive Bewertung durch das Institut für Qualität und Wirtschaftlichkeit im Gesundheitswesen (IQWIG), gefolgt von der Einholung von Sachverständigengutachten und Expertenanhörungen und letztendlich der G‑BA-Beschluss im November 2018, das Screening zu implementieren [16, 17]. Seit August 2019 ist die SCID-Erkrankung im Rahmen des erweiterten Neugeborenenscreenings (Stand 01.01.2023: 16 Krankheitsgruppen) enthalten und steht somit allen in Deutschland geborenen Kindern zur Verfügung.

Dieser Artikel fasst die Grundlagen des Screenings, den Ablauf der Bestätigungsdiagnostik sowie erste Ergebnisse seit Einführung im August 2019 zusammen.

## Biologische Grundlagen für ein Neugeborenenscreening auf SCID

Patienten mit SCID leiden an einem angeborenen schweren T‑Zell-Mangel. Bei allen klassischen Formen von SCID und auch syndromalen Grunderkrankungen mit T‑Zell-Defizienz sind die Ausreifung von T‑Zellen im Thymus und die damit verbundene Entwicklung sogenannter naiver T‑Zellen gestört [2]. Bei der physiologischen Entwicklung der T‑Zelle entstehen im Rahmen der Reifungsprozesse episomale DNA-Moleküle, die als „T cell receptor excision circles“ (TRECs) bezeichnet werden. Die Quantifizierung von TRECs dient als Surrogatparameter für die naive T‑Zell-Zahl im Blut und somit für die Thymusfunktion bei Geburt. Ein Fehlen bzw. ein deutlich verringerter Wert dieser TRECs ist suggestiv für einen schweren Mangel an naiven T‑Zellen. Laborchemisch lassen sich die TRECs mittels Realtime quantitativer PCR (rt-qPCR) aus einer herkömmlichen Trockenblutkarte bestimmen. Die Methodik kann somit unkompliziert in bestehende Neugeborenenscreenings inkorporiert werden [18]. Eine direkte Bestimmung der T‑Zell-Zahl ist mit dieser Methode nicht möglich, so dass bei einem auffälligen Screening-Befund aus der Trockenblutkarte eine Bestätigungsdiagnostik aus peripherem Vollblut mit einem anderen Verfahren (Durchflusszytometrie) folgen muss (siehe unten).

## Ablauf des Screenings und der Bestätigungsdiagnostik

Das Neugeborenenscreening auf SCID erfolgt in Deutschland im Rahmen des erweiterten Neugeborenenscreenings [19]. Die Blutentnahme auf eine Trockenblutkarte findet normalerweise im Alter zwischen 36 und 72 Lebensstunden statt. Im Falle einer Frühgeburtlichkeit unter der 32. Schwangerschaftswoche (SSW) muss im korrigierten Alter von 32 SSW ein Zweitscreening erfolgen. Für die SCID-Erkrankung können gemäß der Kinder-Richtlinie (*Richtlinie des Gemeinsamen Bundesausschusses über die Früherkennung von Krankheiten bei Kindern*) quantitative und semiquantitative PCR-Protokolle eingesetzt werden [20]. Neben zertifizierten Medizinprodukten kommerzieller Hersteller können auch *Inhouse*-Verfahren genutzt werden. Aufgrund der heterogenen Diagnostik sind *Cut-off-*Werte für TRECs laborindividuell festgelegt. Auffällige TREC-Werte werden entweder als (i) vermindert (≤ *Cut-off-*Wert) bzw. als (ii) stark erniedrigt/ganz fehlend klassifiziert. Letztere werden in Anlehnung an die englischsprachige Terminologie innerhalb des Algorithmus und in der Literatur als *Urgent Positive* bezeichnet.

Gemäß der Kinder-Richtlinie werden auffällige TREC-Werte analog anderer auffälliger Screening-Befunde an den Einsender (in der Regel Geburtshelfer oder Kinderärzte) berichtet [16, 20]. Diese informieren dann die Eltern über den Befund und die Notwendigkeit einer unmittelbaren Bestätigungsdiagnostik. Die Bestätigungsdiagnostik, die neben der klinischen Evaluation auch eine weiterführende durchflusszytometrische Untersuchung zur Bestimmung der T‑Zell-Zahl umfasst, soll nach auffälligem SCID-Screening in sogenannten spezialisierten immunologischen Einrichtungen erfolgen. Hierbei handelt es sich um ein Netzwerk von Institutionen, die über eine langjährige klinische und diagnostische Expertise im Umgang mit schwer immundefizienten Kindern (insbesondere an SCID erkrankten Kindern) verfügen. Nach Bekanntgabe des G‑BA-Beschlusses zum SCID-Screening haben daher die in der Behandlung und Diagnostik von SCID-Kindern federführenden Fachgesellschaften API, DGKJ, DGNS und die Gesellschaft für Pädiatrische Onkologie und Hämatologie (GPOH) ein Beurteilungsverfahren zur Identifikation geeigneter Einrichtungen festgelegt. Der Beurteilungs- und Auswahlprozess wurde von internationalen Experten begleitet.

**Figure 1:**
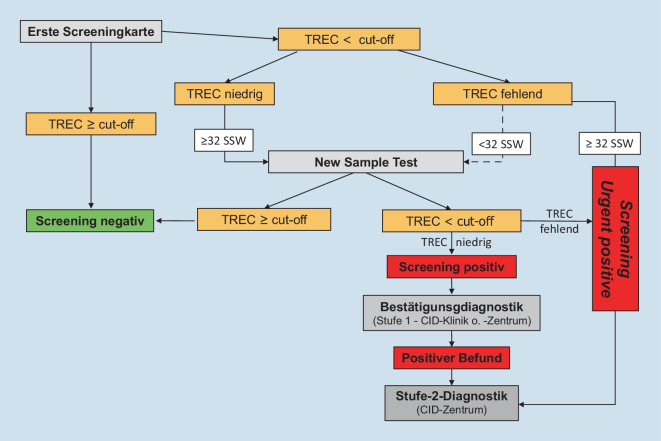

Hieraus entstand in Deutschland ein Netzwerk sogenannter Combined-Immunodeficiency(CID)-Kliniken und CID-Zentren [21]. Diese führen eine definierte *Level‑1*-Bestätigungs- bzw. Ausschlussdiagnostik durch und leiten den Patienten ggf. an ein CID-Zentrum für die *Level‑2*-Diagnostik weiter. CID-Zentren initiieren prophylaktische und ggf. therapeutische Maßnahmen und sind insbesondere für die Durchführung einer kurativen HSZT am Standort vorgesehen. Ebenso koordinieren sie im Bedarfsfall die Organisation einer Thymustransplantation bzw. Gentherapie. Neugeborene mit einem erniedrigten TREC-Wert werden an eine CID-Klinik oder an ein CID-Zentrum überwiesen. Im Falle stark erniedrigter bzw. vollständig fehlender TRECs sollte die Überweisung direkt an ein CID-Zentrum erfolgen, damit protektive Maßnahmen und die *Level‑2*-Diagnostik zeitnah eingeleitet werden können (Abb. 1). Die Strukturanforderung einer CID-Klinik bzw. eines CID-Zentrums bzw. die in der *Level-1-* und *Level-2-*Diagnostik erhobenen Parameter sind in Tab. 1 aufgeführt. Der Algorithmus und eine Liste der CID-Kliniken und CID-Zentren ist online^1^ veröffentlicht, zudem steht eine Telefonhotline^2^ für medizinisches Personal zur Verfügung.

**Table 1:** 

	CID-Klinik	CID-Zentrum
	Bestätigungsdiagnostik bei verminderten TRECs	Bestätigungsdiagnostik bei *Urgent Abnormal*/abnormalen TRECs
Personelle Voraussetzungen	Mind. 2 in Vollzeit an der Klinik beschäftigte Fachärzte für Kinder- und Jugendmedizin mit Erfahrung in pädiatrischer Immunologie	Mind. 2 in Vollzeit an der Klinik beschäftigte Fachärzte für Kinder- und Jugendmedizin mit Erfahrung in pädiatrischer Immunologie
	24-Stunden-Erreichbarkeit eines Arztes der Einrichtung als Kontakt für die Screening-Labore (inkl. Wochenenden und Feiertage)	24-Stunden-Erreichbarkeit eines Arztes der Einrichtung mit Erfahrung in pädiatrischer Immunologie (auch im Ruf‑, Bereitschafts- oder Hintergrunddienst) als Kontakt für die Screening-Labore (inkl. Wochenenden und Feiertage)
	Unmittelbare Terminvergabe zur Erhebung der (Familien‑)Anamnese und klinischen Untersuchung	Unmittelbare Terminvergabe zur Erhebung der (Familien‑)Anamnese und klinischen Untersuchung
Strukturelle Voraussetzungen	**Labor**	**Labor**
	Immunologisches Labor vor Ort oder in Kooperation	Immunologisches Labor vor Ort
	Befähigung zur Durchführung der *Level‑1*-Diagnostik	Befähigung zur Durchführung der *Level-1-* und *Level‑2*-Diagnostik
	Teilnahme an Ringversuchen	Teilnahme an Ringversuchen
	**Register**	**Register**
	Aktive Meldung in das ESID-/PID-NET-Register	Aktive Meldung in das ESID-/PID-NET-Register
		Meldung in das SCETIDE-Register und/oder
		Meldung in das PRST/EBMT-Register
		**Räumlichkeiten**
		Möglichkeit der räumlichen Isolation von SCID-Patienten im Einzelzimmer mit Schleuse oder Überdruck (ggf. auch auf einer Intensivstation)
		Vorhandensein einer pädiatrischen Stammzelltransplantationseinheit in der Einrichtung
		**Patienten**
		Behandlung von Patienten mit schwerer T‑Zell-Lymphopenie mit HSZT
		Behandlung von Patienten mit anderen Immundefekten mit HSZT
		Patienten mit primären Immundefekten in regelmäßiger ambulanter Betreuung
Verpflichtungen	Positiv gescreente Patienten mit auffälliger Bestätigungsdiagnostik müssen an ein CID-Zentrum zur Planung einer weiterführenden Therapie (i. d. R. Stammzelltransplantation) zugewiesen werden	Meldung positiv gescreenter Patienten mit gesicherter T‑Zell-Lymphopenie in das ESID-/PID-NET-Register
	Rückmeldung der Befunde der Bestätigungsdiagnostik an das Screening-Labor	Meldung positiv gescreenter Patienten an den Neugeborenenscreening-Rat der API
		Meldung aller SCID-Patienten an das SCID-SZT-Register
		Rückmeldung der Befunde der Bestätigungsdiagnostik an das Screening-Labor
Labordiagnostik	Verfügbarkeit jederzeit werktags	Verfügbarkeit jederzeit werktags
	Befundung werktags binnen 24 h	Befundung werktags binnen 24 h
	Teilnahme an Ringerversuchen (Immunphänotypisierung)	Teilnahme an Ringerversuchen (Immunphänotypisierung)
	**Parameter des „*Level 1*“**	**Parameter des „*Level 1*“**
	Differentialblutbild	Differentialblutbild
	Immunglobuline IgM, IgG, IgA, IgE	Immunglobuline IgM, IgG, IgA, IgE
	Quantitative Immunphänotypisierung mit	Quantitative Immunphänotypisierung mit
	T‑Zellen (CD3/CD4/CD8)	T‑Zellen (CD3/CD4/CD8)
	T‑Zell-Naivität (CD45RA/CD45R0)	T‑Zell-Naivität (CD45RA/CD45R0)
	B‑Zellen (CD19)	B‑Zellen (CD19)
	NK-Zellen (CD3/CD16/CD56)	NK-Zellen (CD3/CD16/CD56)
		**Parameter des „*Level 2*“**
		Quantitative Immunphänotypisierung mit
		T‑Zellen (CD3/CD4/CD8)
		T‑Zell-Naivität (CD45RA CCR7)
		*Recent Thymic Emigrants* (CD4/CD31/CD45RA)
		aß- und yd-T-Zellen (CD3/aßTCR/ydTCR)
		B‑Zellen (CD19)
		NK-Zellen (CD3/CD16/CD56)
		Bei Nachweis relevanter T‑Zell-Zahlen:
		Analytik auf maternale T‑Zellen
		Lymphozytenproliferationstest (PHA und/oder Anti-CD3/Anti-CD3/CD28)
		TCRVbeta-Repertoire
		Einleitung weiterführender Spezialdiagnostik: Adenosindesaminase (ADA), Purin-Nukleosid-Phosphorylase (PNP), Sanger und/oder *Next Generation Sequencing*. HIV-Ausschlussdiagnostik bei Mutter und Kind (PCR)

*API* Arbeitsgemeinschaft Pädiatrische Immunologie, *CID* kombinierter Immundefekt, *ESID European Society for Immunodeficiencies, HIV* humanes Immundefizienz-Virus, *HSZT* hämatopoetische Stammzelltransplantation, *PID-NET* Netzwerk für primäre Immundefekte, *PRST/EBMT* Pädiatrisches Register für Stammzelltransplantation – European Society for Blood and Marrow Transplantation. Register zur Erfassung der HSZT Behandlungsergebnisse auf deutscher (PRST) und europäischer (EBMT) Ebene, *SCETIDE Stem Cell Transplantation for Immunodeficiencies in Europe Registry *(internationales Register zur Erfassung der HSZT-Behandlungsergebnisse bei angeborenen Immundefekten in Europa), *SCID* schwerer kombinierter Immundefekt, *TRECs* „T cell receptor excision circles“

In Deutschland liegt die Geburtenrate in den letzten Jahren weitestgehend konstant zwischen 700.000–800.000 Neugeborenen pro Jahr [22]. Die große Mehrheit der Kinder wird im Rahmen des erweiterten Neugeborenenscreenings mittels Trockenblutkarte in einem der 11 Screening-Labore untersucht (vgl. 2020: laut amtlicher Statistik 773.144 Kinder geboren, davon hatten 769.320 ein Screening, das entspricht einer Screening-Rate von 99,51 % aller Neugeborenen; [23]). Positive Befunde für alle Zielerkrankungen werden von den Laboren an die DGNS im Rahmen der Qualitätssicherung gemeldet. Eine Infrastruktur zum systematischen longitudinalen Tracking von Neugeborenen mit positivem Screening-Befund, d. h. eine Erfassung der Bestätigungsdiagnostik und Outcome-Daten nach erfolgten Therapien, ist bedauerlicherweise weder für SCID noch für andere Zielerkrankungen Bestandteil des Neugeborenenscreenings in Deutschland. Dies zeigt sich z. B. eindrucksvoll beim Hörscreening, welches auch im Neugeborenenalter durchgeführt wird: Für > 40 % der Kinder mit auffälligem Screening gibt es keine Information zum klinischen Verlauf, insbesondere ob die Diagnose der Hörminderung bestätigt werden konnte. Nur einzelne Bundesländer (z. B. Bayern) erfassen diese Informationen zum Verlauf [24].

Die API versucht, dieses organisatorische Defizit des SCID-Neugeborenenscreenings zu kompensieren, indem Patienten mit auffälligem SCID-Screening und anschließend gesicherter Diagnose in wissenschaftliche Immundefektregister eingeschlossen werden. Hierunter sind insbesondere das Nationale Register für primäre Immundefekte (PID-NET) innerhalb des Registers der *European Society for Immunodeficienci*es (ESID) bzw. das multizentrische Therapieregister Schwere Kombinierte Immundefekte (SCID-SZT) mit besonderem Schwerpunkt der Therapie von SCID-Patienten zu nennen. Die AG Screening der API evaluiert zudem in 6 monatlichen Abfragen die Bestätigungsdiagnostik in den CID-Kliniken und -Zentren bzw. den Einschluss in die Studien.

## Bisherige Ergebnisse des Screenings

Erste Zwischenergebnisse des Screenings wurden vor Kurzem veröffentlicht [25]. Hierzu wurden Screening-Daten (DGNS-Report und PID-NET/ESID-Register bzw. SCID-SZT-Register) von August 2019 bis Dezember 2021 evaluiert. In dieser Zeit hatten 1.878.985 Neugeborene eine TREC-Untersuchung mittels Trockenblutkarte im erweiterten Neugeborenenscreening. 99,9 % (1.877.057) zeigten normale Werte. 1443 Neugeborene hatten einen TREC-Wert unterhalb des lokalen *Cut-off-*Wertes, hiervon 175 Kinder (davon 58 mit einem Gestationsalter von < 32 SSW) mit einem fehlenden/stark erniedrigten TREC-Wert. Eine zweite Karte wurde von 1268 Neugeborenen angefordert und bei 1182 von ihnen durchgeführt. Hierbei ist zu betonen, dass ein Großteil dieser Patienten entweder ein Gestationsalter < 32 SSW aufwiesen (*n* = 589) und/oder sich auf einer neonatologischen Intensivstation (*n* = 389) befanden. Informationen zu 229 Patienten mit erfolgter Bestätigungsdiagnostik konnten durch die Abfragen der API gewonnen werden. 66 hatten eine normale Durchflusszytometrie und sind somit als falsch-positiv zu werten. In den meisten Fällen konnte die Ursache nicht ermittelt werden, bei einigen Trockenblutkarten wird eine Inhibition der PCR aufgrund der Verwendung von heparinisiertem Blut (teilweise auch in Unkenntnis der Beschichtung der Blutentnahmekapillare) vermutet [26]. 35 Patienten zeigten eine sekundäre Ursache einer T‑Zell-Lymphopenie (z. B. Vorgeburtlichkeit, gastrointestinale Lymphangiektasien, maternale Immunsuppression). Insgesamt konnten bis Februar 2022 im Rahmen des Screenings 88 Patienten mit einer primären T‑Zell-Lymphopenie diagnostiziert werden. Diese Patienten wurden basierend auf klinischen, immunologischen und genetischen Befunden in 3 Gruppen eingeteilt – die Klassifikation folgt im Wesentlichen den Kriterien des *Primary Immune Deficiency Treatment Consortium* (*PIDTC*) von 2014 [27]: 25 der 88 identifizierten Patienten wurden als klassischer SCID eingestuft. 10 der 88 Patienten zeigten einen „*Leaky-SCID*“-Phänotyp bzw. ein Omenn-Syndrom. Beim *Leaky SCID* handelt es sich um eine Sonderform, bei welcher aufgrund hypomorpher Mutationen T‑Zellen nicht vollständig fehlen. Beim Omenn-Syndrom sind diese fehlgeleiteten autoreaktiven T‑Zellen für die klinische Ausprägung der Immundysregulation verantwortlich und können teilweise auch stark erhöht sein. 8 der 88 auffälligen Screening-Ergebnisse wurden als idiopathisch/reversibel bzw. unklar gewertet. Die meisten Patienten zeigten dennoch eine niedrige T‑Zell-Zahl, die sich im Verlauf erholte.

Ein Großteil der Patienten (46/88) hatte eine syndromale Grunderkrankung, meist eine Mikrodeletion 22q11 (DiGeorge-Syndrom, *n* = 20) oder einen anderen Thymusanlagedefekt. Die Inzidenzen verteilen sich wie folgt: 1:54.000 Neugeborenen für SCID, *Leaky SCID* und Omenn-Syndrom, 1:41.000 für syndromale Grunderkrankungen mit T‑Zell-Defizienz. Insgesamt ergibt sich eine Inzidenz für schwere T‑Zell-Lymphopenien von 1:21.000 Neugeborenen.

20 von 25 SCID-Patienten wurden bereits innerhalb der ersten 4 Monate stammzelltransplantiert, 2 weitere innerhalb der ersten 6. 2 Patienten waren zum Zeitpunkt der Auswertung noch jünger als 4 Lebensmonate und befanden sich in Vorbereitung auf eine HSZT. Bei einem SCID-Patienten mit radiosensitiver Erkrankung wurde die HSZT für Ende des ersten Lebensjahres terminiert.

Im letzten Follow-up im April 2022 waren 20 der 21 transplantierten Patienten am Leben (Follow-up 1–27 Monate, Median 12,7 Monate). Ein Patient starb vor der HSZT an einer Parainfluenzainfektion. 2 Patienten starben kurz nach der Geburt an den Folgen eines schwersten und bereits *in utero* einsetzenden Omenn-Syndroms. Bei einer Patientin mit ADA-SCID wurde eine Gentherapie geplant. 5 Patienten mit Thymusanlagedefekten und vollständigem Fehlen von naiven T‑Zellen erhielten eine Thymustransplantation am Great Ormond Street Hospital in London. Die meisten der Patienten mit syndromaler Grunderkrankung und T‑Zell-Dysfunktion werden durch immunologische Fachambulanzen betreut und erhalten supportive Therapien. 7 dieser Patienten verstarben an nichtimmunologischen bzw. nichtinfektiologischen Komplikationen, insbesondere schweren Herzfehlern.

Durch Auswertung der API-Daten konnten außerdem 2 SCID-Patienten identifiziert werden, die trotz des Screenings entweder nicht erkannt oder nicht rechtzeitig einer Bestätigungsdiagnostik zugeführt wurden. Für eine Patientin mit ADA-SCID wurde initial fälschlicherweise ein normaler TREC-Wert berichtet. Die Patientin entwickelte in den ersten Lebenswochen eine interstitielle Lungenerkrankung und im Alter von 4 Monaten eine ausgeprägte Lymphopenie. Die ursprüngliche Trockenblutkarte wurde erneut untersucht und zeigte bei wiederholten Messungen komplett fehlende TRECs. Retrospektiv wurde eine Kreuzkontamination bei der Probenverarbeitung im Screening-Labor vermutet; eine methodische Umstellung des Pipettierprotokolls erfolgte umgehend. Ein weiterer Patient mit Purin-Nukleosid-Phosphorylase(PNP)-Defizienz (und verminderten TRECs in der initialen Karte) wurde erst zeitverzögert einer Kontrolluntersuchung (Untersuchung einer Zweitkarte) zugeführt. Aufgrund des Fehlens eines systematischen Trackings von Screening-Befunden fiel der ausbleibende Befundrücklauf nicht auf. Der Patient wurde daher nicht weiterverfolgt und die Eltern stellten das Kind nach Auftreten von klinischen Symptomen im Alter von 9 Monaten vor. Zu diesem Zeitpunkt lag bereits ein Epstein-Barr-Virus-(EBV-)positives Lymphom vor, an welchem der Patient kurz darauf verstarb. Der vormals auffällige Screening-Befund war den Eltern nicht bekannt.

## Diskussion

Das Neugeborenenscreening auf SCID wird in Deutschland mittlerweile seit 4 Jahren durchgeführt und stellt mit einer Screening-Rate von etwa 800.000 Neugeborenen jährlich das größte SCID-Screening-Programm in Europa dar [23]. Aufgrund infrastruktureller Vorgaben und der föderalen Struktur Deutschlands erfolgt die Testung wie für alle anderen Zielerkrankungen des erweiterten Neugeborenenscreenings in 11 regionalen Screening-Laboren. Im Gegensatz zu anderen europäischen Ländern endet das Neugeborenenscreening in Deutschland mit der Befundübermittlung an den Einsender und ggf. dem Auftrag zur Durchführung einer Bestätigungsdiagnostik [28–32]. Diese wird durch die federführenden Fachgesellschaften abgestimmt. Im Falle des SCID-Screenings wurde die Identifikation von geeigneten immunologischen Einrichtungen (CID-Kliniken und -Zentren) durch die API koordiniert [28]. Die API koordiniert auch den Einschluss Betroffener in wissenschaftliche Register und wertet diese in 6‑monatlichen Abständen aus. Eine aktuelle Publikation konnte zeigen, dass die Abfragen innerhalb des API-Netzwerkes eine große Datenkonsistenz mit den von den Screening-Laboren gemeldeten Daten (DGNS-Report) zeigen [23, 25]. Mit 35 identifizierten SCID-/*Leaky-SCID*-Patienten (August 2019 und Februar 2022) war die Inzidenz von schweren T‑Zell-Mangelerkrankungen mit 1:54.000 höher als durch vergangene Studien bzw. retrospektive Erhebungen vermutet. Die Inzidenz von SCID und schweren kongenitalen T‑Zell-Lymphopenien in Deutschland ist vergleichbar mit anderen Regionen Europas und Nordamerikas, in denen ein prospektives SCID-Screening eingeführt wurde. Erfreulicherweise zeigen die aktuellen API-Auswertungen auch, dass die Patienten früh identifiziert und meist auch erfolgreich therapiert werden. Im Vergleich zu einer retrospektiven Studie vor Einführung des Screenings konnte die Mortalität signifikant gesenkt werden [33].

Ferner ist bemerkenswert, dass in der deutschen SCID-Screening-Kohorte die Anzahl genetisch ungeklärter Fälle sehr niedrig ist (4 % bei SCID, 12 % bei allen kongenitalen T‑Zell-Lymphopenien). Wir vermuten, dass sich dieses unter anderem mit zunehmend verbesserten diagnostischen Sequenziertechnologien (Screening-Ergebnisse Deutschland 2019–2022 vs. Screening-Ergebnisse USA 2010–2017) erklären lässt; grundsätzlich ist der einfachere Zugang zu schneller genetischer Diagnostik in Deutschland aber sicherlich auch ein wichtiger Faktor.

2 Patienten mit auffälligem Neugeborenenscreening zeigten bereits *in utero* ein schwerstes Omenn-Syndrom und waren somit schon als Neugeborene klinisch auffällig. Trotz unverzüglicher supportiver Therapie verstarben sie vor Einleitung einer HSZT. Bis auf ein Kind mit fehlender Nachverfolgung (Trackingfehler) bei positivem Screening (PNP-SCID) erhielten alle im Screening identifizierten SCID-Patienten rechtzeitig eine adäquate Versorgung, die vor allem neben der Initiierung krankheitsspezifischer Maßnahmen (medikamentöse Prophylaxen, Isolation, Einleitung einer Enzymersatztherapie bei ADA-SCID) auch die zeitige Planung einer HSZT umfasste.

In den wenigen Fällen, bei denen die erkrankungsspezifischen therapeutischen Maßnahmen in Deutschland nicht zur Verfügung stehen, erfolgte die zeitgerechte Vorbereitung und Überweisung an andere europäische Kliniken zur Durchführung anderer zelltherapeutischer Maßnahmen (z. B. Gentherapie bei ADA-SCID oder Thymustransplantation bei Thymusanlagedefekten). Bislang ist die Überweisung in andere EU-Länder und das Vereinigte Königreich (trotz Austritt aus der EU) nach entsprechendem Antrag für Krankenversicherte aus Deutschland möglich. Es ist sicherlich zu berücksichtigen, dass auch andere europäische Länder nationale Screening-Programme implementieren und so der Zufluss auf die wenigen europäischen Behandlungsorte (z. B. in London oder Mailand) zunehmen wird. Ebenfalls zeigt der Trend der letzten Jahre, dass viele Gentherapieprogramme aufgrund unterschiedlicher, teils kommerzieller Hürden zunehmend eingestellt werden. Der breite Zugang zu diesen Therapieformen, idealerweise in akademischen Institutionen, bleibt eine wichtige gesundheitspolitische Aufgabe in den nächsten Jahren [4].

Aus den aktuellen Auswertungen geht aber leider auch hervor, dass zwei SCID-Patienten trotz Screenings nicht rechtzeitig identifiziert werden konnten und daher verstarben. Ein Fall wurde retrospektiv als technischer Prozessierungsfehler der Probe im Screening-Labor erkannt. Die Methode konnte umgehend angepasst werden, so dass ähnliche Fälle in Zukunft unwahrscheinlich sind. Ein zweiter Fall zeigt die aus unserer Sicht kritischen Probleme eines fehlenden systematischen Befundtrackings des deutschen Neugeborenenscreenings auf. Der Befund eines kontrollbedürftigen Screenings wurde verschickt, aufgrund fehlender systematischer Nachverfolgung und Erfassung (z. B. durch Trackingeinrichtungen auf Landes- oder Bundesebene) fiel jedoch nicht auf, dass die vorgesehene Befundkontrolle ausblieb. Die SCID-Diagnose wurde daher erst bei Auftreten klinischer Symptome gestellt. Ein systematisches und longitudinales Tracking von Kindern mit auffälligem Screening-Befund könnte dies wirkungsvoll verhindern; politische Vorgaben oder Vorschläge zur Einführung und Refinanzierung von Strukturen existieren hierzu auf nationaler Ebene derzeit jedoch nicht. Nur wenige Regionen und Länder (z. B. Bayern) haben „Screening-Zentren“, die solchen Trackings nachkommen, bereits installiert [34].

Trotz dieser verbleibenden Hürden zeigen die vorläufigen Daten von API und DGNS, dass das TREC-Neugeborenenscreening in Deutschland erfolgreich angelaufen ist. Auch wenn Langzeitdaten noch nicht verfügbar sind, wird die Zielerkrankung SCID rechtzeitig und sicher identifiziert. Die kurative HSZT erfolgt deutlich früher, mit besseren Voraussetzungen und damit einer höheren Überlebenswahrscheinlichkeit. Auch andere Ursachen eines schweren angeborenen T‑Zell-Mangels („bonus targets“) – wie z. B. syndromale Grunderkrankungen mit T‑Zell-Lymphopenie – werden früh erkannt und profitieren wirkungsvoll von der Einleitung prophylaktischer Maßnahmen. Eine umfängliche Einbindung des Screenings innerhalb eines Trackingsystems und die systematische Analyse bzgl. Diagnostik und Behandlungsverlauf aller pathologischen TREC-Befunde innerhalb eines Registers bleiben wichtige zukünftige Ziele.

## Footnotes

1 https://kinderimmunologie.de/?page_id=262 Letzter Zugriff 01.06.2023.

2 https://kinderimmunologie.de/?page_id=258 Letzter Zugriff 01.06.2023.
